# Combining Distance Matrices on Identical Taxon Sets for Multi-Gene Analysis with Singular Value Decomposition

**DOI:** 10.1371/journal.pone.0094279

**Published:** 2014-04-14

**Authors:** Melanie Abeysundera, Toby Kenney, Chris Field, Hong Gu

**Affiliations:** Department of Mathematics and Statistics, Dalhousie University, Halifax, Canada; National Institute of Environmental and Health Sciences, United States of America

## Abstract

We present a simple and effective method for combining distance matrices from multiple genes on identical taxon sets to obtain a single representative distance matrix from which to derive a combined-gene phylogenetic tree. The method applies singular value decomposition (SVD) to extract the greatest common signal present in the distances obtained from each gene. The first right eigenvector of the SVD, which corresponds to a weighted average of the distance matrices of all genes, can thus be used to derive a representative tree from multiple genes. We apply our method to three well known data sets and estimate the uncertainty using bootstrap methods. Our results show that this method works well for these three data sets and that the uncertainty in these estimates is small. A simulation study is conducted to compare the performance of our method with several other distance based approaches (namely SDM, SDM* and ACS97), and we find the performances of all these approaches are comparable in the consensus setting. The computational complexity of our method is similar to that of SDM. Besides constructing a representative tree from multiple genes, we also demonstrate how the subsequent eigenvalues and eigenvectors may be used to identify if there are conflicting signals in the data and which genes might be influential or outliers for the estimated combined-gene tree.

## Introduction

Phylogenetic analyses with individual genes will often result in conflicting topologies for the same set of taxa in the estimated evolutionary trees [1]. The goal of multi-gene analysis is to combine information across several genes in such a way that a single tree representation of the relationship for the same set of taxa is obtained from multiple genes. There are two popular methods for combining information across multiple genes: concatenation (supermatrix method) and consensus (supertree method). In the former the aligned sequences from multiple genes are concatenated and a phylogenetic analysis is performed on the concatenated sequences [1]–[7]. This approach assumes all genes share a common evolutionary history which is not necessarily valid and may result in an incorrect estimate of the species tree [1], [8], [9]. In the latter a separate tree is inferred for each gene and a single tree is estimated by consensus [10]–[12]. Except several more recent computationally intensive methods which are based on either bootstrap gene trees or posterior distributions of gene trees [13]–[15], most algorithms for this method ignore the uncertainties in the estimated gene trees and thus underestimate the variation for inferred species phylogeny. More recently several new maximum likelihood species tree inference methods that explicitly model both the mutational and coalescent effects were proposed and achieved better accuracy [16]–[19], however these methods can only deal with trees with small number of taxa due to the computational complexity.

When a large number of taxa are examined, distance-based methods are useful tools for building a starting tree to be further refined by maximum likelihood methods. Distance-based methods typically combine the distances inferred from multiple genes to obtain a summary measure of distances for a common set of taxa. A few methods to do this have been proposed. The average consensus supertree (ACS) [20] computes an average distance matrix using all the scaled input path-length matrices. ACS provides a least-squares estimate of the input path-length matrices. Note that the aim of ACS was to compute a consensus tree from a set of input trees and thus the path-length matrices in ACS were derived from input trees which could be first estimated from different genes. Bevan, Lang and Bryant [21] use a weighted least squares approach to estimate the evolutionary rates of individual proteins and thereby estimate a representative distance for each taxa pair from multiple genes. In their method, estimated distances are weighted according to their level of uncertainty. The weights are based on a given substitution model [22]. The super distance matrix (SDM) [23] computes a single scale coefficient for each distance matrix, as well as external branch length correction coefficient for each taxon inside each considered gene so that the linearly transformed pairwise distances across genes are close in a weighted least squares sense. A combined-gene distance matrix is then given by the weighted average of the transformed distance matrices with the weights given as the number of characters of each multiple sequence alignment. A version of SDM, named SDM*, that estimates only the distance matrix scale coefficients (i.e. every external branch length correction coefficient set to zero) is also described in [23]. Criscuolo and Michel [24] further studied the impact of other weights when using SDM* to combine the three distance matrices directly estimated from each codon position, especially weights based on the arboricity coefficient [25]. Recently, Abeysunderra, Field and Gu [26] introduced the minimum coefficient of variation squared (MinCV) criterion for selecting a single scale coefficient to weight each distance matrix. Like SDM*, the MinCV attempts to bring the distance matrices as close together as possible by minimizing the coefficient of variation squared between the distance matrices.

In this paper we consider an alternative method for deriving multiple gene tree estimates based on singular value decomposition (SVD). Stuart, Moffett and Leader [27] applied SVD to tetrapeptide frequency matrices to select the most informative biomolecular sequence characteristics from which to estimate evolutionary distances. Here we are interested in extracting the consistent signal present in the dissimilarity or distance matrices for each gene and using this information to obtain a combined-gene tree. The method should work best when the gene trees are the same and the branch lengths are proportional between genes, but should be robust to slight deviations from these assumptions. This method can be applied using any taxonomic distance measure. The method is illustrated using both the common scaling spectral covariance (SpCov) based dissimilarities [28] and Jones-Taylor-Thornton (JTT) [29] based distances. A simulation study is carried out to compare the performance of SVD with that of SDM, SDM* and ACS97. Combined-gene trees for primate, nematode and chloroplast data sets are estimated and the variability about the estimated trees is determined using bootstrap samples.

## Methodology

We begin by transforming the dissimilarities or distances for all pairs of taxa into p-vectors where 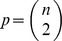 for 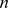 taxa. The p-vectors for a set of 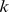 genes are combined into a single matrix 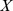. The rows of 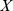 correspond to genes and the columns to taxa pairs. That is, 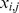 is the pairwise dissimilarity or distance of the 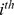 gene and the 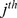 taxa pair, where 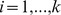 and 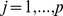.

Let 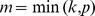. The matrix 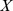 can be decomposed as follows.
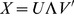
(1)where 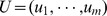 is an orthogonal 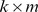 matrix containing the non-zero eigenvectors of the matrix 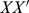, where 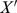 is the transpose of 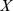, and 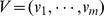 is an orthogonal 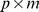 matrix containing the non-zero eigenvectors of 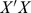 and 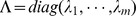 is the diagonal 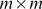 matrix of singular values. The column vectors in 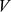 give the directions of the principal components 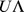 [30].

The distances between pairs of taxa in a phylogenetic tree can be expressed as a linear combination of the estimated branch lengths between those taxa for a given tree topology [31]. For a set of 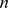 taxa, any particular set of 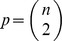 pairwise distances representing a tree topology can be expressed as a set of 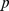 equations involving 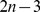 variables or branches, which in turn can be expressed as a topology matrix 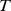 multiplied by a branch length vector 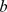. That is, for a vector of estimated distances for gene 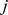, say 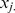, we get the following matrix equation.
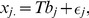
(2)where 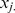 denotes a column vector formed from the 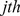 row of the 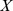 matrix, 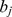 is a 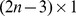 vector of branch lengths for gene 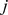 and 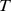 is a 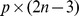 topology matrix. If the distances 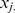 are true distances for tree 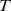, then 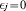 in equation (2). For one gene, we can think of this as regressing the pairwise distances estimated from the gene on the variables corresponding to the branches in topology 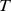. Finding the tree topology is equivalent to finding the 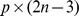 topology matrix 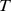 which corresponds to the smallest errors. The popular methods are based on least squares (LS) [32] or weighted least squares (WLS) criteria [33]. A natural generalization of the LS criterion for 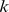 genes is then to find the tree topology 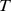 such that the total squared error for 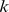 genes are minimized, that is:
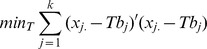
Assuming topology 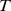 is the true topology, if there is a consistent signal among genes about the underlying tree and noise in the data is small (the noise here mainly comes from imperfect estimation of the pairwise distances, and can include both bias from model misspecification, and stochastic variance) the rows of matrix 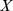 should belong to or be close to a subspace spanned by the columns of 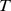. Furthermore, in practice, the estimated branch lengths 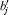 from different rows of 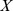 are often proportional to each other. Under this proportionality assumption, denote the unit length direction given by the proportional vectors 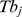 as 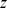, then 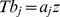 with a scale vector 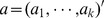. The above criterion can be re-written as
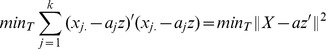
Note that all vectors without 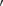 are defined as column vectors, and 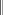 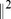 denotes the sum of squares of all elements in the matrix.

The first eigenvectors 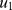 and 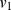 in the SVD of 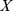 solves the problem 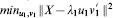 subject to 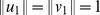. Thus a natural LS estimate for 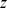 is 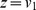 and 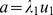. A single tree representation for multiple genes can be obtained using the first right eigenvector of the SVD of the pairwise distance matrix 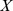. A new 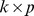 distance matrix, 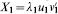 is the distance matrix with reduced noise. Note that the pairwise distances for each gene in 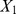 are all scaled versions of 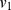 and thus describe the same tree topology. Hence, this equates to using de-noised distances to estimate a single representative topology for multiple genes for a given set of taxa. Since 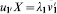, the elements in 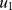 gives the weights such that 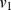 can be interpreted as a weighted sum of pairwise distance vectors of different genes provided by the rows of 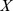.

If the underlying trees of different genes are not consistent or there is large noise in the data, the direction 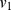 will reflect the signal of the majority of the genes with its direction disturbed by the outliers. Note that our proportionality assumptions on branch lengths correspond to there being no lineage-specific rate heterogeneity beyond gene-specific rates. If this assumption does not hold, but the underlying trees for all genes are the same, the topology estimated by SVD is still consistent. Since if all the gene distances lie on some hyperplane, associated with a particular topology, then the first eigenvector of SVD which is a linear combination of the gene distances will also lie on that hyperplane.

Geometrically, each row of 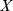 is a vector in 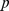 dimensional space. Because the elements in 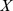 are pairwise distances and are all non-negative, these vectors all start from the origin and point in the positive quadrant. The first right eigenvector 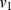 is a vector from the origin pointing towards the center of all these vectors. This property assures that all elements in vectors 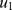 and 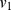 are positive and this positivity property is unique for these two eigenvectors because matrices 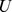 and 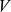 are both orthogonal. Furthermore, if the row vectors in 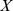 all satisfy the distance properties, then 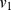 also satisfies the distance properties.

The major source of variability from the origin for rows of X lies on the direction 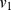. The percentage of information captured by 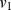 is 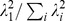. If this number is close to 1, then there is a consistent signal among genes and noise in the data is small. If this number is much less than 1, then it is an indication that the underlying trees of different genes are not consistent or there is large noise in the data, in which case, further investigation on the subsequent eigenvalues and eigenvectors is informative in presenting the additional information beyond noise. More specifically, the row vectors in 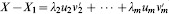 are all in the orthogonal space of vector 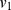. The dominant information in 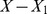 can be easily graphically displayed in the coordinate system spanned by the vectors 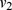 and 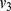, i.e. we can plot 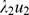 versus 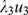 as a diagnostic plot to find if there is any important pattern in the data that is beyond only noise. The origin of this diagnostic plot is the position 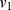 is pointing at, and each point on this plot corresponds to a gene, with its relative position to the origin showing the error of this gene relative to the principal phylogenetic signal in 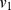. If the data show clustered patterns, we will know which genes are grouped together and with which group the principal signal is mainly associated. Re-analyses of different groups might be informative in this situation. If the data show several outliers, then re-analysis without the outliers should be performed and the results can be compared to the full data analysis.

Many methods for calculating pairwise distances for a gene will result in larger distances for the fast evolving genes. For example JTT based distances [29] belong to this type. Typically the variances of the pairwise distance estimates for these fast evolving genes are much larger. In order to avoid the undue influences of these fast evolving genes on the computed direction 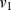, it is recommended that the rows in matrix 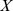 be normalized before performing SVD in such situations. That is, we divide the entries 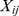 by 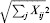, so that each row of the 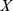 matrix has Euclidean norm 1. Geometrically, the normalization corresponds to projecting all 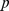 vectors in the rows of 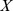 to a 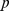 dimensional unit sphere. Since the sum of squared eigenvalues is equal to the trace of the matrix 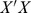, the total sum of squared eigenvalues is 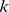 after the rows in matrix 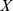 are normalized. If the variances of the pairwise distance estimates are not associated with the lengths of the rows in matrix 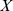, or the pairwise distance estimates have been normalized in the process of its calculation, such as the SpCov-based dissimilarities [28], then performing SVD directly on the 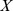 matrix is preferable. In either case, it should be noted that the first eigenvector 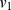 is normalised, so provides the relative scales of the pairwise distances, but does not give an overall distance for the tree. The scale is not important for reconstructing the tree topology.

Typically the computational complexity of SVD for a 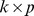 matrix is 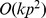 if 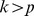 and 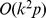 if 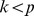. Thus the algorithmic complexity of our method for distance matrices built from 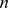 taxa and 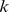 genes is 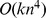 or 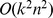, whichever is smaller. This is comparable to the practical computational complexity of SDM which is slightly less than 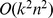 in the consensus setting here [23] (and much better than the theoretical complexity of SDM, which is 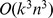).

## Simulations

We will quantify the ability of the SVD approach for inferring accurate trees by comparing its performance with several other distance-based approaches in the consensus setting where distance matrices combined have identical taxa. Criscuolo, et al. [23] have implemented two methods that combine rescaled distance matrices into a unique one: SDM*, and ACS97 [20], and showed that distance-based trees inferred from these two methods are less accurate than expected during simulations. Based on their simulation results, Criscuolo, et al. [23] have implemented a more parameter-rich method (i.e. SDM) that leads to better results. We will compare the performances of 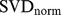 which is SVD on the normalized pairwise distance matrices, SVD which is SVD on the original pairwise distance matrices, SDM, SDM* and ACS97.

Data are simulated using the same simulation protocol used by [23] with some modification for our case. We will base our simulation design on the Chloroplast data set as described in the *results* section. There are 22 taxa and 25 genes with the sequence lengths of genes the same as those in the Chloroplast data set. We first generate 100 random 22-taxon trees using the standard Yule-Harding process via the R8S program [34] as was done by [35], and [23]. To generate deviations from the clock-like trees, every branch length is multiplied by 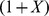, where 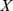 follows an exponential distribution with expectation 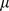. The 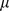 value represents the extent of deviation and was identical within each tree but different from tree to tree and equal to 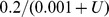, with 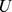 being uniformly drawn from 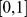. The smaller the 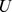, the larger the 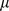 and the larger the deviation from the molecular clock. This gives us 100 simulated “true” trees.

Next, to simulate the evolution of different genes corresponding to each of these simulated “true” trees, 25 “gene” trees were generated from each of these 100 trees. Note that instead of simulating DNA sequences as in [23], we need to simulate amino acid sequences. Thus our tree lengths need to be re-scaled so that the branch lengths can be interpreted as the expected number of amino acid changes per site. To do this, for each randomly generated topology, we randomly draw 25 numbers from the distribution of the total tree lengths of 56 chloroplast genes published by [36] and assign these tree lengths to the 25 “gene” trees. These tree lengths range from 0.130 to 6.557, so the simulated data includes genes with very different evolutionary rates. Thus each branch in each “gene” tree is multiplied by its assigned tree length and divided by the length of the original tree. This was repeated for the 100 simulated trees to obtain 2500 “gene” trees. For each of these 2500 trees, 100 sequences were simulated in SEQ-GEN [37] using the cpRev substitution matrix for proteins encoded by chloroplast DNA [38].

Since the sites of the simulated sequences are independent, it is more sensible to use the JTT-based distances instead of SpCov-based dissimilarities. Thus we only calculate the JTT-based distance matrices. This means that there is some misspecification in the model, since the trees were simulated using the cpRev model. For each of the 100 “true” trees, we have 100 replicates. For each replicate, we constructed a combined distance matrix using each of the methods SVD, 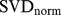, SDM, SDM* and ACS97, and estimated a tree from this combined distance using BioNJ. For each method we therefore had 10000 estimated trees. The Robinson-Fould distances [39] between the “true” trees and the estimated trees are summarized in Table 1.

**Table 1:** Robinson-Fould distances between the estimated trees based on methods SVD, 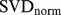, SDM, SDM* and ACS97 and the “true” trees for 10000 cases.

RF	SVD	SVD_norm_	SDM	SDM*	ACS97
0	5505	6669	6653	6660	6755
2	3374	2845	2855	2852	2786
4	931	444	448	447	419
6	168	41	43	40	40
8	21	1	1	1	
10	1				

From Table 1, we can see for JTT distances, the SVD method on the normalized distance matrices greatly outperforms the SVD method on the original distance matrices. We also see that 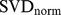 has comparable performance to the SDM, SDM* and ACS97, with ACS97 slightly better, but not statistically significantly so, than the other methods.

## Results

Three different data sets are used in this paper to illustrate our method. We begin with an exploratory analysis on a non-controversial primate data set. We then apply our methods to the nematode data set published in [40] and a chloroplast data set considered by [41], [42] and [43]. These data sets have also been analysed by [26]. The Genbank accession numbers for all the data can be found in the Supplementary material. Sequences were aligned using ClustalW [44] in Bioedit [45] and the characters containing gaps were discarded, so that the sequences for each gene have the same number of characters. Short genes were removed from the data sets, because the SpCov method (see below) does not perform well for genes with fewer than about 30 amino acids.

The method is illustrated using both the common scaling spectral covariance (SpCov) based dissimilarities [26], [28] and Jones-Taylor-Thornton (JTT) [29] based distances. The SpCov based dissimilarity is a measure converted from the similarity measure for a pair of sequences with its value scaled between 0 and 1. The SpCov based similarity is a summary measure of the common periodicities between two categorical sequences. The method relies on the smoothed Fourier transform of the cross-spectra between two sequences. A high covariance at a given frequency signifies a common periodicity between two sequences at that frequency. In the calculation, a common scaling for both sequences is chosen for each frequency so that the squared spectral covariance at each frequency attains the maximum possible value. The SpCov method of sequence comparison does not assume any particular evolutionary model, but instead is a distance method based on spectral analysis which takes into account correlations among sequence sites. To assess the variabilities of the estimated trees, in the case of the SpCov-based dissimilarities the bootstrap method uses block permutation samples, which generates bootstrap samples of the data by random permutations of the blocks of the original sequences. This ensures the dependence structure between the sites of protein sequences is partially preserved in the bootstrap samples [26]. Since the JTT model of evolution assumes independence of sites, variability in the trees based on these distances is estimated from bootstrap samples obtained by sampling the individual sites within sequences with replacement. JTT-based distances are estimated using the protdist program from the PHYLIP package [46]. Bootstrap samples used with the JTT model are obtained using the seqboot program from the PHYLIP package [46]. The rows of the JTT-based distance matrices are all normalized before performing SVD, while SVD is directly applied on the SpCov-based dissimilarity matrices since each dissimilarity measure has already been scaled. The tree building methods BIONJ [47] and FITCH [33] are used to estimate trees from the combined-gene distances obtained with the SVD method.

### Primate Data Set

**Figure 1:**
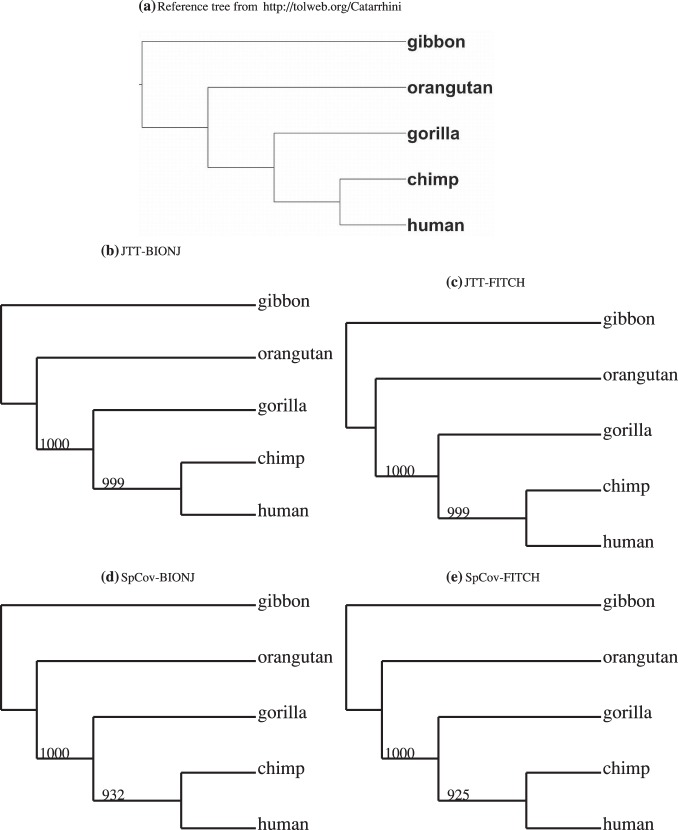
Primate data: reference tree from http://tolweb.org/Catarrhini (a) and the majority-rule consensus trees by the SVD method estimated from 1000 bootstrap samples with JTT-BIONJ (b); JTT-FITCH (c); SpCov-BIONJ (d); SpCov-FITCH (e).

The method is first applied to the simple primate data. The primate data set has five taxa: *Hylobates agilis* (gibbon), *Pongo pygmaeus* (orangutan), *Gorilla gorilla* (gorilla), *Pan trogodytes* (chimp) and *Homo sapiens* (human). It consists of thirteen mitochondrial protein-coding genes. The phylogeny for the primate data set is well established with the species tree shown as in Figure 1 (a). However based on a phylogenetic analysis of 23,210 DNA sequence alignments from human, chimpanzee, gorilla, orangutan, and gibbon, for about 23% of the nuclear genes in the genome, human and chimpanzee are not placed together as sister taxa (with clades of human-gorilla and chimpanzee-gorilla occurring with about the same frequency). The explanation for this is incomplete lineage sorting [48]. Since the data set we are analysing consists of mitochondrial genes, we would expect the genes to follow the established species tree.

The four combined-gene trees from BIONJ and FITCH methods, using the first right eigenvectors of the SVD of the 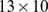 matrices of JTT distances and SpCov-based dissimilarities, all correspond to the reference tree topology shown in Figure 1 (a).

**Figure 2:**
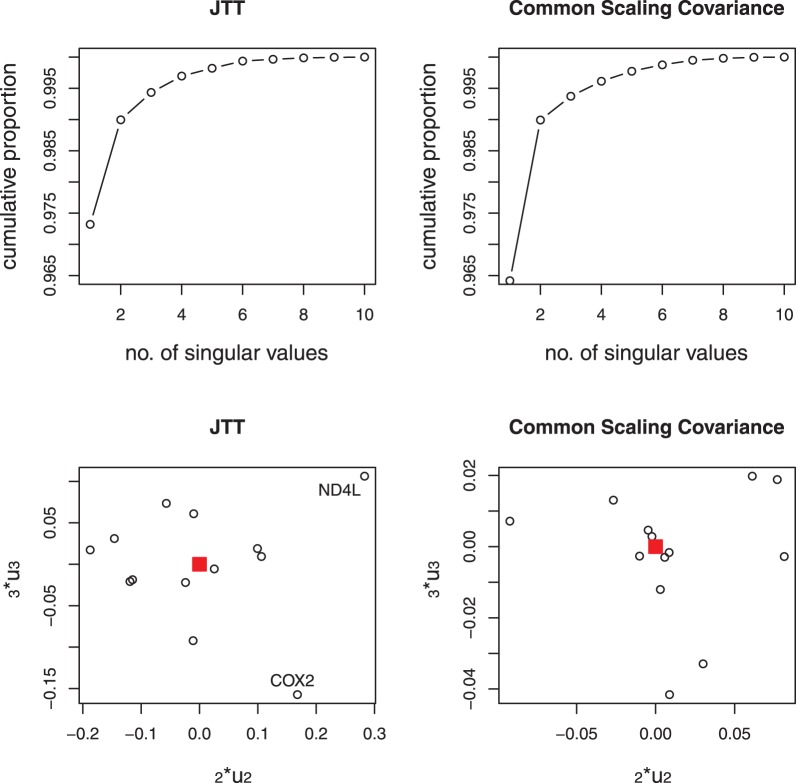
Primate data. Top panels show cumulative proportion of the sum of the squared singular values of the SVD of JTT-based distances (left) and the SpCov-based dissimilarities (right); bottom panels show the diagnostic plots of 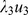 versus 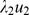 from SVD of JTT-based distances (left) and the SpCov-based dissimilarities (right). The solid squares in the bottom panels label the positions of the directions of 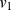.

The top panels of Figure 2 show the cumulative proportion of the squared singular values in the SVD of the JTT-based distances and the SpCov-based dissimilarities. One can see that the first squared singular values make up about 97% and 96% of the sum of the squared singular values for the JTT-based distances and the SpCov-based dissimilarities, respectively. These high percentages mean that there is a consistent signal among genes and noise in the data is small. The sum of the first three squared singular values account for more than 99% of the sum of the squared singular values for both the JTT-based and SpCov-based dissimilarities. The bottom panels of Figure 2 show the diagnostic plots of 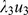 versus 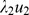 from SVD of the JTT-based distances and SpCov-based dissimilarities. The solid squares in the bottom panels label the positions of the directions of 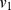 in these two cases. The slight outlier genes are ND4L and COX2 for JTT-based distances. Re-analysis without these outlier genes result the same tree topology as the reference tree topology. Thus these outliers are not influential to the estimated combined-gene tree topology.

For the trees estimated from the JTT-based distances the bootstrap support was evaluated from 1000 bootstrap samples obtained by sampling individual characters from sequences with replacement. A majority-rule consensus tree is computed using the CONSENSE programme in PHYLIP [46]. The majority-rule consensus trees derived from the JTT-based distances obtained with the BIONJ and FITCH tree building methods are shown in Figure 1 (b)–(c). To evaluate the bootstrap support of the estimated trees from SVD of the SpCov-based dissimilarities, 1000 bootstrap samples are generated using the block permutation method with block size 14. The SVD method is applied to each of them and the majority-rule consensus trees derived from the SpCov-based dissimilarities and obtained with the BIONJ and FITCH tree building methods are shown in Figure 1 (d)–(e). It can be seen that the combined-gene trees estimated from the first right eigenvectors of the SVD for both JTT-based distances and SpCov-based dissimilarities strongly support the reference tree. For the JTT-based distances, 99.9% of the bootstrap trees recover the reference tree. For the SpCov-based dissimilarities approximately 93% of the bootstrap permutation trees recover the reference tree with both BIONJ and FITCH. Gorilla and human branch as sister taxa with chimp as an outgroup for this clade in a small proportion (i.e. 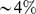) of the bootstrap trees. A few bootstrap trees also erroneously place human as outgroup to a chimp and gorilla clade (i.e. 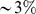). The estimated uncertainties of the SVD based combined-gene trees obtained with both distance methods appear to be very small for this data set. These results are consistent with the results shown in Figure 2, that the signals across genes are consistent and thus the uncertainty of the estimates is small.

### Nematode Data Set

**Figure 3:**
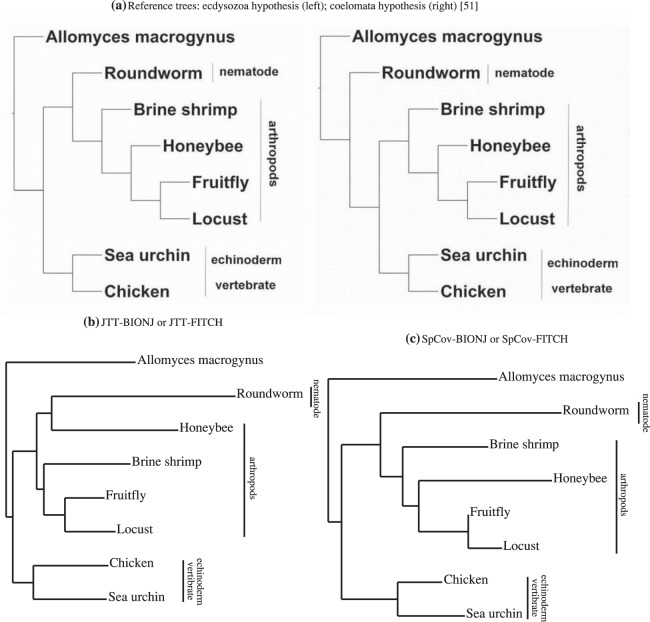
Nematode data. Reference topologies under the ecdysozoa hypothesis and coelomata hypothesis (top) [51]; Combined-gene trees estimated from first right eigenvectors of the SVD with JTT-BIONJ or JTT-FITCH (b); with SpCov-BIONJ or SpCov-FITCH (c).

The method is next applied to the more difficult nematode data set which is known to have problems with both long-branch attraction and compositional bias [40]. The data set consists of twelve mitochondrial protein-coding genes common to eight animals. There has been some debate regarding the placement of the nematodes in relation to other animals and two rival theories, i.e. the ecdysozoa hypothesis and the coelomata hypothesis, have formed. Figure 3(a) shows the reference trees under both these hypotheses. Based on a phylogenetic analysis of 16S ribosomal DNA sequences, Aguinaldo, et al. [49] first proposed a clade of nematodes and arthropods as that in the ecdysozoa hypothesis. Later a phylogenetic analysis on the complete genomes of 11 taxa was carried out by [50] and again found strong support for the ecdysozoa hypothesis. Both of these two analyses chose a more slowly evolving nematode and excluded the fast-evolving sequences of *Caenorhabditis elegans*, which is used in our analysis, as a representative nematode. Their results indicated a strong relationship between the nematode and the arthropods. However, Blair et al. [51] found strong support for the coelomata hypothesis based on their analyses on 100 individual protein data sets consisting of four taxa. Another genome-wide analysis using a type of rare genomic change robust to long branch attraction and taxon sampling again found strong support for the coelomata hypothesis [52]. Philippe, Brinkmann and Lartillot [53] argued that strong support for the coelomata theory was due to sparse taxon sampling. They analysed 146 genes on a sample of 35 taxa and found strong support for the ecdysozoa hypothesis.

Figure 3(b) and Figure 3(c) show the combined-gene trees obtained using the first right eigenvectors of the SVD of the 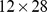 matrices of JTT-based distances and SpCov-based dissimilarities with BIONJ tree building method. The tree topologies obtained with FITCH method are the same as that from BIONJ method in both cases. The combined-gene tree estimated from the SpCov-based dissimilarities agrees with the reference tree under the ecdysozoa hypothesis shown in Figure 3(a). The combined-gene tree estimated from JTT-based distances corresponds to the topology found using concatenated sequences presented in [40], with the roundworm (nematode) and honeybee being erroneously grouped together as sister taxa. As observed in [26], using the SpCov-based dissimilarities which are based on structure could better correct this erroneous pairing of roundworm and honeybee.

**Figure 4:**
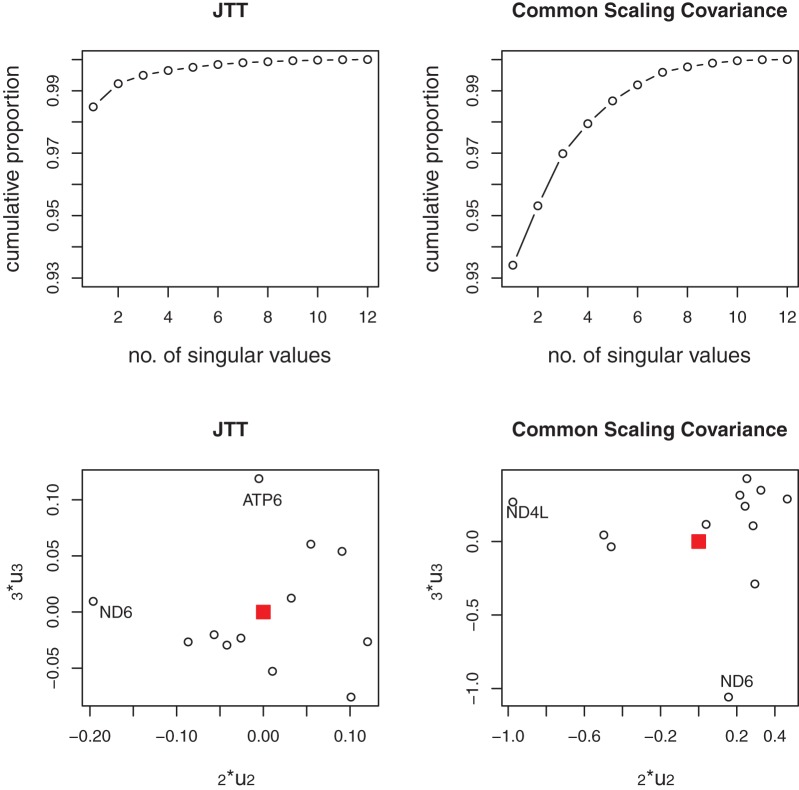
Nematode data. Top panels show cumulative proportion of the sum of the squared singular values of the SVD of JTT-based distances (left) and the SpCov-based dissimilarities (right); bottom panels show the diagnostic plots of 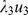 versus 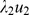 from SVD of JTT-based distances (left) and the SpCov-based dissimilarities (right). The solid squares in the bottom panels label the positions of the directions of 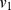.

The top panels of Figure 4 show the cumulative proportion of the squared singular values among the sums of all squared singular values. For the nematode data set, the first squared singular values make up 98.5% and 93% of the total, and the sums of the first three squared singular values make up 99.5% and 97% in the SVD of JTT-based distances and SpCov-based dissimilarities, respectively. These high percentages mean that there is a consistent signal among genes and noise in the data is small again for this data set. The bottom panels of Figure 4 show the diagnostic plots of 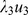 versus 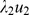 from SVD of the JTT-based distances and SpCov-based dissimilarities. There are no obvious outlier genes although we have labelled a couple of genes which are slightly further away from the center of the data. Given the small percentage of the squared singular values in the subspace spanned by the second and the third right eigenvectors, we expect no change of the tree topologies estimated in the re-analyses when omitting these labelled genes. That is indeed the case. Omitting these observations, the resulting trees correspond to the same trees shown in Figure 3(b) and Figure 3(c) respectively.

**Figure 5:**
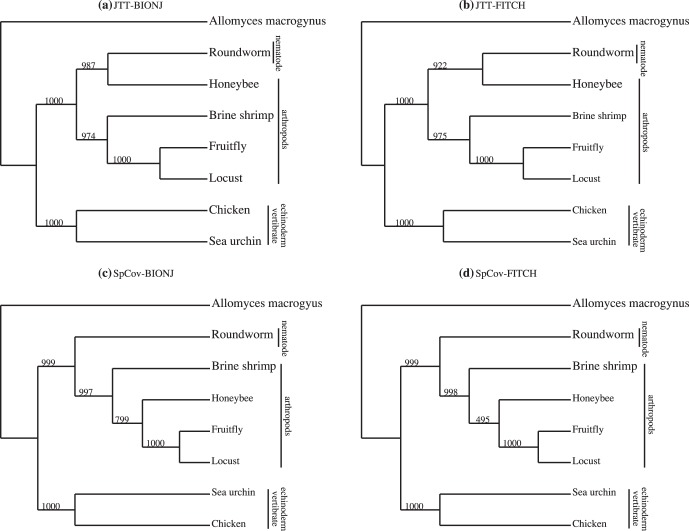
Nematode data. The majority-rule consensus tree by the SVD method estimated from 1000 bootstrap samples with JTT-BIONJ (a); JTT-FITCH (b); SpCov-BIONJ (c) and SpCov-FITCH (d).

The estimated combined-gene topologies have strong bootstrap support, though there is some variability within the estimated topologies of the arthropod clade. The BIONJ and FITCH majority-rule consensus trees derived from 1000 bootstrap trees for the JTT-based distances and the SpCov-based dissimilarities are shown in Figure 5. Under both distance methods the bootstrap trees indicate that the variation with regard to the relative placement of vertebrates, nematode and arthropods is small. Variability in the placement of honeybee in particular is greater in trees estimated with FITCH than those estimated with BIONJ. The estimated trees derived from the JTT-based distances have strong bootstrap support. The JTT-based bootstrap trees group honeybee and roundworm as sister taxa in 98.7% of BIONJ trees and 92.2% of FITCH trees. However, separation of honeybee and roundworm occurs in more than 99% of the trees estimated from the SpCov-based dissimilarities. 80% of BIONJ trees and 50% of FITCH trees obtained with the SpCov-based dissimilarities recover the reference tree. The bootstrap trees obtained with the SpCov-based dissimilarities recover the reference tree with greater frequency than the bootstrap trees obtained with the JTT-based distances. However, the estimated variation in the arthropod clade for the combined-gene tree obtained with SpCov-based dissimilarities is relatively high, particularly for the honeybee.

### Chloroplast Data Set

**Figure 6:**
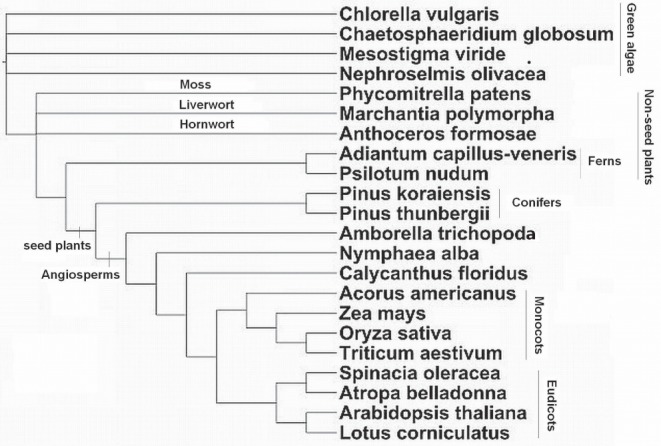
Chloroplast data. Reference tree topology with 22 taxa [43].

As a final example, we apply the method to the larger chloroplast data set [42] which has 22 taxa and 25 genes. For this data, the debate has been mainly over the placement of *Amborella trichopoda* within the angiosperms. The majority of analyses place *Amborella* as a sister taxon to the rest of the angiosperms [54]–[56]. However, in some cases an *Amborella+Nymphaea* clade was found, and placed as sister to the rest of the angiosperms [57], and [26] found a clade of *Calycanthus floridus, Amborella trichopoda* and *Nymphaea alba* as sister to the rest of the angiosperm clade. Goremykin et al. [58] presented an alternative topology with the monocots as the most basal lineage of the angiosperms, and *Amborella* in a clade with *Calycanthus*, which was refuted by [59], [60] and later [61] showed that model misspecification and long branch attraction was the cause of the monocot-first topology. The most recent and comprehensive analyses [62]–[64] support placing *Amborella trichopoda* as sister to the rest of the angiosperms. This tree is now widely accepted. An important point to note about the controversy here is that the papers that place *Amborella* as basal use parsimony or maximum likelihood, while the distance methods have placed *Amborella* in a clade with *Nymphaea*. Since our method is a distance method, it may be subject to the same inclination to place *Amborella* in a clade with *Nymphaea*, and possibly *Calycanthus*. The reference tree for the chloroplast data [43] is shown in Figure 6.

**Figure 7:**
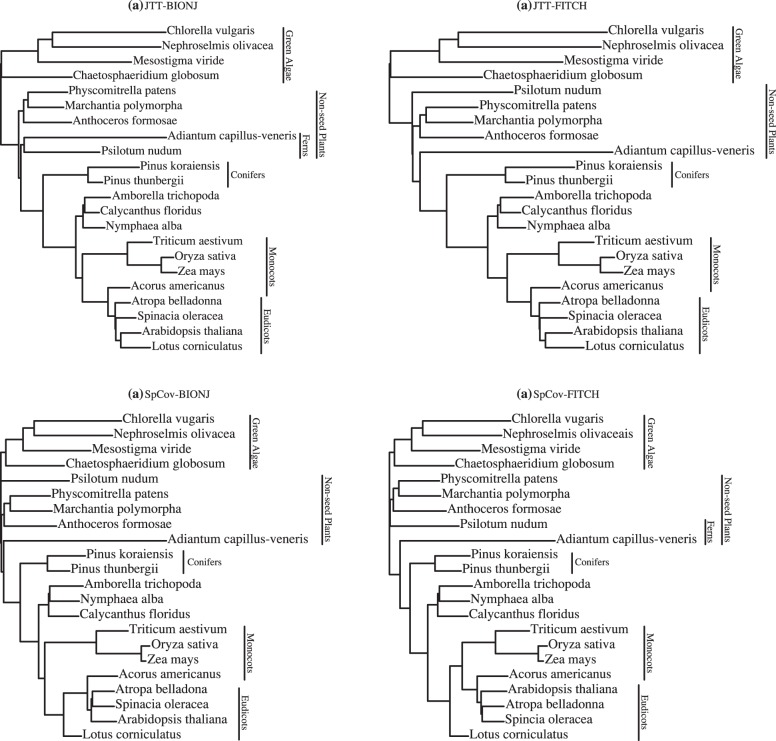
Chloroplast data. Combined-gene trees estimated from first right eigenvectors of the SVD with JTT-BIONJ (a); JTT-FITCH (b); SpCov-BIONJ (c) and SpCov-FITCH (d).

Figure 7 shows the combined-gene trees obtained using the first right eigenvectors of the SVD of the 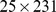 matrices of JTT-based distances and SpCov-based dissimilarities computed with the BIONJ and FITCH methods. Separation of taxa into the major groups of green algae, non-seed plants, conifers and angiosperms shown in the reference tree in Figure 6 is recovered by all these combined-gene trees. The controversies between these trees are all within the subtrees of non-seed plants and angiosperms. Among these four combined-gene trees, the JTT-BIONJ tree is the closest to the reference tree. The differences between the reference tree and JTT-BIONJ tree are all within the clade of angiosperms. The JTT-BIONJ tree places a clade of *Calycanthus floridus, Amborella trichopoda* and *Nymphaea alba* as the most basal lineage in the angiosperm clade, as given in [26]. It also places one monocot, *Acorus americanus*, as a sister taxon to the eudicots. The JTT-FITCH tree is the same as JTT-BIONJ within the angiosperms clade, but the JTT-FITCH tree erroneously separates the two ferns, *Psilotum nudum* and *Adiantum capillus-veneris*. This separation of two ferns is again observed in both the SpCov-BIONJ and SpCov-FITCH trees. Within the angiosperms clade, the SpCov-BIONJ and SpCov-FITCH trees both place a clade of *Amborella trichopoda, Nymphaea alba* and *Calycanthus floridus* as the most basal lineage and place one monocot, *Acorus americanus*, among the eudicot clade. In addition, the SpCov-FITCH tree also places *Lotus corniculatus* as the most basal lineage in the clade of eudicots and monocots.

**Figure 8:**
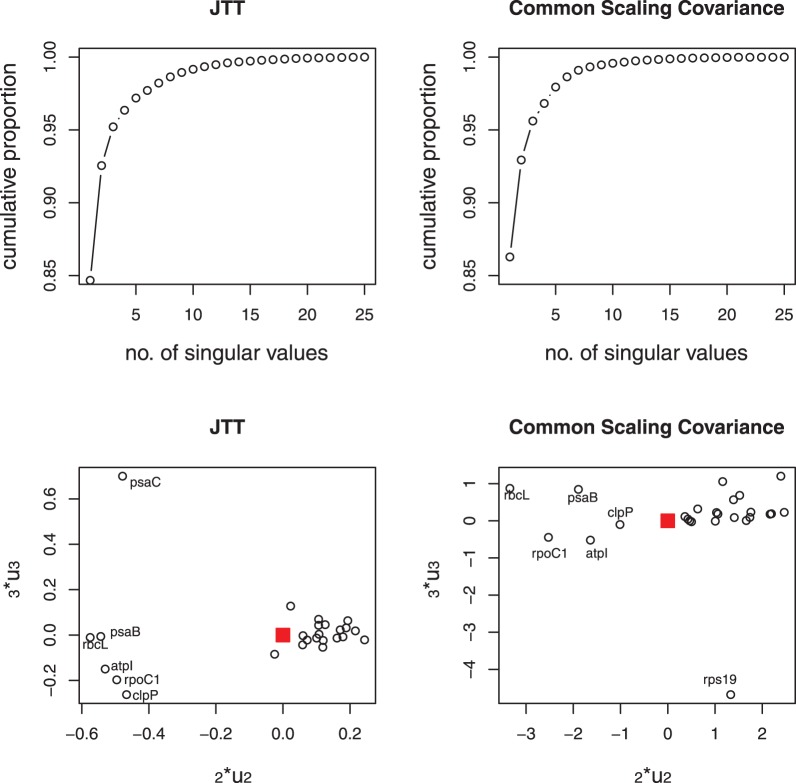
Chloroplast data. Cumulative proportion of the sum of the squared singular values of the SVD of JTT-based distances (top left) and the SpCov-based dissimilarities (top right); Diagnostic plots of 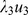 versus 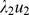 from SVD of JTT-based distances (bottom left) and the SpCov-based dissimilarities (bottom right). The solid squares in the bottom panels label the positions of the directions of 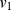.

The existence of such controversies can be explained by the following diagnostic plots. The top panels of Figure 8 show the cumulative proportion of the squared singular values among the sums of all squared singular values. For this data set, the first squared singular values only make up 85% and 86% of the total, and the sums of the first three squared singular values make up 95% and 96% in the SVD of JTT-based distances and SpCov-based dissimilarities, respectively. The bottom panels of Figure 8 show the diagnostic plots of 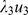 versus 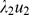 from SVD of the JTT-based distances and SpCov-based dissimilarities. In the JTT-based distance diagnostic plot, points show an interesting pattern. There are two tight clusters of genes with one outlier (gene *psaC*). The first cluster consists of genes *atpI, clpP, psaB, rbcL* and *rpoC1*; the rest of the 19 genes belong to the second cluster with the direction 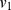 close to the second cluster. In the diagnostic plot from SpCov-based dissimilarities, the same five genes *atpI, clpP, psaB, rbcL* and *rpoC1* are clearly separated from the rest with a different outlier, (*rps19*). The direction 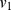 is in the middle of these two clusters. The two outliers are different, but both are outlying on the 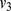 direction. Considering the third squared eigenvalues only make up 2% of the total in both cases, the influence of this outlier should not be very large. The five genes 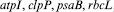, and 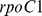 were previously shown to be important in placing *Amborella trichopoda, Nymphaea alba* and *Calycanthus floridus* as a sister clade to the remainder of the angiosperms, in the covariance-based chloroplast tree [26]. Re-analysis using the group of 19 genes given in the JTT-based distance diagnostic plot resulted a very similar BIONJ tree as the JTT-BIONJ tree in Figure 7. The only differences are that the clade of two ferns changes position with the clade of the other three non-seed plants, and a clade of monocots (*Triticum aestivum, Oryza sativa* and *Zea mays*) is placed as sister to the rest of the angiosperm clade, with the clade of *Calycanthus floridus, Amborella trichopoda* and *Nymphaea alba* grouped together with *Acorus americanus*. Re-analysis with just the five genes *atpI, clpP, psaB, rbcL* and *rpoC1* doesn’t result a very sensible tree, but it does place the clade of *Calycanthus floridus, Amborella trichopoda* and *Nymphaea alba* very far away from most of the other angiosperm species, which might explain why these five genes are important in placing *Amborella trichopoda, Nymphaea alba* and *Calycanthus floridus* as sister to the rest of the angiosperm clade for the analysis with the full set of genes.

**Figure 9:**
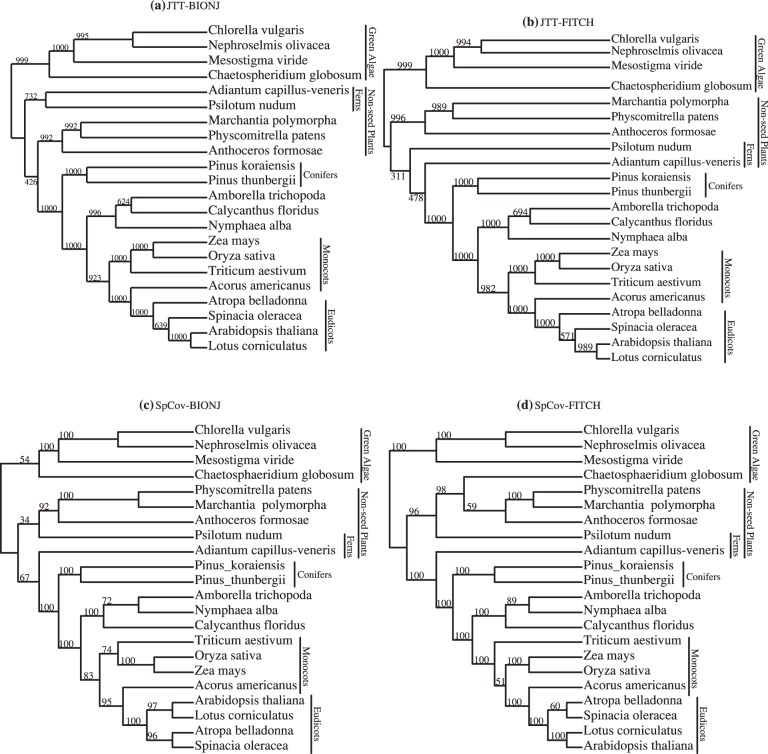
Chloroplast data. The majority-rule consensus trees by the SVD method estimated from 1000 bootstrap samples with JTT-BIONJ (a); JTT-FITCH (b) and from 100 bootstrap samples for SpCov-based dissimilarities with SpCov-BIONJ (c) and SpCov-FITCH (d).

These controversies are all again shown in the consensus trees in Figure 9. Due to the size of this data set, the bootstrap support of the combined-gene tree using SpCov-based dissimilarities was estimated from 100 bootstrap samples rather than 1000 bootstrap samples, since calculating SpCov dissimilarities is time consuming. All the bootstrap trees obtained with both distance methods separate green algae, non-seed plants, conifers and angiosperms, with a clade of *Amborella trichopoda, Nymphaea alba* and *Calycanthus floridus* as sister to the rest of the angiosperm clade. Within the *Amborella trichopoda, Nymphaea alba* and *Calycanthus floridus* clade, the majority of bootstrap trees from JTT-BIONJ and JTT-FITCH place *Calycanthus floridus* and *Amborella trichopoda* as sister taxa and the majority of bootstrap trees from SpCov-BIONJ and SpCov-FITCH place *Amborella trichopoda* and *Nymphaea alba* as sister taxa. The variation in the estimated trees is fairly small with regards to the placement of most of taxa including some of those in the angiosperm clade. However, surprisingly the two taxa with the greatest variation regarding their placement in the estimated combined-gene trees are the two ferns, *Psilotum nudum* and *Adiantum capillus-veneris*. Among these bootstrap trees, Only 73% of the JTT-BIONJ, 41% of the JTT-FITCH, 33% of the SpCov-BIONJ and 0% of the SpCov-FITCH trees recover the clade of two ferns seen in the reference tree in Figure 6.

## Discussion

The results obtained from the application of the singular value decomposition method for obtaining combined-gene phylogenetic trees indicate that the performance of the method at estimating the reference tree topologies is comparable with the best other distance methods. The variability of the estimates obtained with this method was found to be mostly small, suggesting that the common signal extracted from the multiple genes using this method is fairly strong relative to noise or conflicting signals. The subsequent eigenvectors of the SVD of the distance matrix may be used as diagnostics to identify outlier genes or groups of genes with conflicting signals. The simulation results confirm that the performance of the SVD method is comparable to other existing similar types of methods. In our view, SVD provides a very simple and useful method for combining the phylogenetic signal as measured by distances across genes, and it has potential to be developed further to achieve better results due to its simplicity.

As shown in the Methodology section, the SVD method provides a generalization of the least squares criterion for multiple genes to combine the phylogenetic signals. This can be easily extended to the weighted least squares criterion if the assumption that the diagonal variance matrices for the pairwise distance estimates across different genes are proportional to each other holds. In this case, denote the inverse of one diagonal variance matrix as 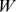, (the proportionality coefficients don’t make any difference in the results), we right multiply the 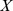 matrix by 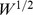 before performing SVD, then we transform the resulting first right eigenvector 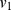 to 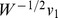 and use 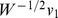 to construct the combined-gene tree. More exploration on this issue is needed before extending SVD to the weighted least squares criterion. The results obtained for the nematode and chloroplast data sets which are known to suffer from long-branch attraction indicate that the SVD method can perform quite well with difficult data sets and provide fairly efficient combined-gene tree estimates.

As we observed in the methodology section, the complexity of the singular value decomposition is 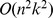 in most cases. In practical terms, this means that the method should scale well, being able to handle 1000 taxa and 1000 genes within a day, even on a standard desktop computer. The speed could be improved even further by only doing a partial SVD, computing only the first few eigenvectors.

We have focused the SVD method on the consensus setting in this article. This is different from the focus of the methods SDM and SDM* which is to construct a distance super-matrix when missing data are present because some genes or species are less represented in databases. However, our method can be easily extended to the cases when missing data are present in the distance matrices. Multiple imputation methods based on the model assumptions or calculating the SVD and imputing the missing values iteratively are two different ways to consider. Currently we are extending the SVD method along these directions.

The SVD method is based on the assumption that most genes have a consistent phylogeny signal and that the estimated branch lengths from different genes are proportional to each other. The second assumption is a strong assumption and may be violated if, for example, the rates of substitution are different for some lineages across different genes. In this case, SVD still gives a consistent topology estimate, but there is potential to improve the efficiency of the method. Instead of finding a vector to combine all phylogenetic signals across genes based on SVD, a better aim is to find the 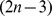 dimensional hyperplane that is associated with a tree topology and with the least error when projecting the distance vectors from different genes on the hyperplane. This will be an interesting subject for subsequent research.

In summary, due to its simplicity, this method has potential to be further developed in multiple ways to be more robust or more efficient, and can have ability to deal with missing values.

## Supporting Information

Table S1 **Primate Genebank numbers.**
(EPS)

Table S2 **Nematode Genebank numbers.**
(EPS)

Table S3 **Chloroplast Genebank numbers.**
(EPS)
